# Amino acid size, charge, hydropathy indices and matrices for protein structure analysis

**DOI:** 10.1186/1742-4682-3-15

**Published:** 2006-03-22

**Authors:** JC Biro

**Affiliations:** 1 Homulus Foundation, San Francisco, CA, USA

## Abstract

**Background:**

Prediction of protein folding and specific interactions from only the sequence (*ab initio*) is a major challenge in bioinformatics. It is believed that such prediction will prove possible if Anfinsen's thermodynamic principle is correct for all kinds of proteins, and all the information necessary to form a concrete 3D structure is indeed present in the sequence.

**Results:**

We indexed the 200 possible amino acid pairs for their compatibility regarding the three major physicochemical properties – size, charge and hydrophobicity – and constructed Size, Charge and Hydropathy Compatibility Indices and Matrices (SCI & SCM, CCI & CCM, and HCI & HCM). Each index characterized the expected strength of interaction (compatibility) of two amino acids by numbers from 1 (not compatible) to 20 (highly compatible). We found statistically significant positive correlations between these indices and the propensity for amino acid co-locations in real protein structures (a sample containing total 34630 co-locations in 80 different protein structures): for HCI: p < 0.01, n = 400 in 10 subgroups; for SCI p < 1.3E-08, n = 400 in 10 subgroups; for CCI: p < 0.01, n = 175). Size compatibility between residues (well known to exist in nucleic acids) is a novel observation for proteins. Regression analyzes indicated at least 7 well distinguished clusters regarding size compatibility and 5 clusters of charge compatibility.

We tried to predict or reconstruct simple 2D representations of 3D structures from the sequence using these matrices by applying a dot plot-like method. The location and pattern of the most compatible subsequences was very similar or identical when the three fundamentally different matrices were used, which indicates the consistency of physicochemical compatibility. However, it was not sufficient to choose one preferred configuration between the many possible predicted options.

**Conclusion:**

Indexing of amino acids for major physico-chemical properties is a powerful approach to understanding and assisting protein design. However, it is probably insufficient itself for complete *ab initio *structure prediction.

## Background

The protein folding problem has been one of the grand challenges in computational molecular biology. The problem is to predict the native three-dimensional structure of a protein from its amino acid sequence. Existing approaches are commonly classified as: (1) comparative modeling; (2) fold recognition; and (3) ab initio methods. The first two methods are knowledge based (database-driven), i.e. some template sequence, which is reliably similar to the target sequence, already exists and the sequence-structure connection is known.

True ab initio approaches rely on Anfinsen's thermodynamic principle [1], which states that protein folding is thermodynamically determined. Amino acid sequences contain all the information necessary to make up the correct three-dimensional structure; that is, given a proper environment, a protein would fold up spontaneously into a conformation that minimizes the total free energy of the system.

None of the protein structure predicting methods perform satisfactorily, which is very frustrating because genome sequencing projects are producing numerous novel coding sequences, and understanding the structure is probably necessary in order to understand the function. Some theoretical considerations suggest that the reason for this inadequacy is probably not methodological and the existing methods perform nearly optimally [2], especially in combination with each other [3].

One possible explanation is that many proteins might have several different but thermodynamically closely-optimal conformations (allosteric variations). This situation is well known from nucleic acid structure predictions [4] where minimal free energy calculations usually produce many possible structure variants. The co-existence of several possible protein configurations is not only possible, but even known and expected, as in substrate-induced change of enzymes [5], and hormone ligand-induced modifications of steroid [6] and peptide [7] hormone receptors.

Another possible reason why protein structure prediction is so difficult is that the scale of the interacting forces is not reliably known; forces acting over short distances (at residue level) might determine completely different structures from forces acting over long distances, and their interaction might involve many neighboring residues (cumulative effects) [8,9]. Our previous studies suggest the importance of interactions at the residue level. We were able to construct a *Common Periodic Table of Codons and Nucleic Acids *that supports co-evolution (stereochemical fitting) of codons and coded nucleic acids [10,11]. We found that codons and coded nucleic acids often localize closely to each other in restriction enzyme-restriction site complexes [12].

The aim of this study was to establish whether it is possible to find statistical correlations between amino acid co-locations (which are determined by the structure) and the physicochemical properties of the co-locating (interacting) amino acid residues.

## Materials and methods

The basic assumption of our method is that the specific protein-protein interaction is governed by well-known, simple rules: opposite charges attract each other; a thin strand might complement a thick strand (convex fits to concave); similar hydrophobicity fits together better than different hydrophobicity. Size, charge and hydropathy are well-known quantitative physicochemical properties and therefore similarities and differences in these properties can be measured and indexed.

We have constructed a series of tentative amino acid interaction matrices to express the similarities and differences between amino acids regarding their physicochemical properties. Each matrix contains 20 × 20 values for 20 amino acids and each value ranges from 1 to 20, where 1 is the lowest (prohibited) and 20 is the highest (favored) probability that two amino acids will interact with each other on the basis of a given physicochemical property.

### Hydrophobe compatibility matrix and index

*Hydropathy *(hydrophobicity vs. hydrophilicity or lipophobicity vs. lipophilicity) is usually characterized by numbers (hydrophobic moments, HM) from -7.5 (Arg) to 3.1 (Ile), whereas *hydrophobicity *is a measure of how strongly the side chains are pushed out of water. The more positive a number, the more the amino acid residue will tend *not *to be in an aqueous environment. Negative numbers indicate hydrophilic side chains, with more negative numbers indicating greater affinity for water [13].

Molecules with similar hydropathy have affinity to each other, they are compatible; molecules with different hydropathy repel each other, and they are not compatible. To express this numerically, we use the hydropathy compatibility index (HCI) and collect these indices (20 × 20) in the matrix. HCIs were calculated using the formula

HCI = 20 - | [HM(A) - HM(B)] × 19/10.6] |

where HM(A) and HM(B) are the hydrophobic moments of the amino acids A and B and HM(Arg)-HM (Ile) = 10.6. This formula gives the maximal index (20) for identical amino acids (closest hydrophobicity) and the minimal value (1) for the two hydrophobically most distant amino acids (Arg and Ile). The "|" indicate absolute values (See 6).

### Charge compatibility matrix and index

Opposite charges attract and similar charges repel each other. The charge of a molecule is pH dependent. It can be characterized by the p*K *values, which are determined for the alpha amino group (N), the alpha carboxy group (C) and the side chain (R, for R-group) for free amino acids. The local environment can alter the p*K*_a _of an R-group when the amino acid is part of a protein or peptide.

A simpler characterization of a molecule's charge properties is the isoelectric point (p*I*), which is the pH at which the overall charge of the molecule is neutral. These values are determined for the entire free amino acid. However, amino acids differ from each other only in side chains. Therefore the p*I *usually reflects differences in the p*K*s of the side chains.

Most amino acids (15/20) have a p*I *very close to 6 so they are regarded as having neutral overall charge; Asp and Glu are negatively charged, acidic (p*I *2.7 and 3.2) and His, Lys, Arg are positively charged, basic (p*I *7.5, 9.7, and 10.7). Only 16/64 codons encode charged amino acids, so the calculated overall frequency of charged amino acids is about 26% and the calculated frequency of charge-determined amino acid-amino acid interactions is 5 × 5/2 of 20 × 20/2, i.e. only 6.25%. The influence of charge on amino acid co-location is therefore much less than the influence of the hydrophobe force.

The intracellular pH is 6.8 while the extracellular pH is 7.4. Those amino acids having lower p*I *than this are negatively charged, those with higher p*I *are positively charged.

For mathematical expression of the size and direction of charge-determined forces, we have constructed the charge compatibility index (CCI) and collected these indexes into a charge compatibility index and matrix (CCI). The formula used to calculate CCI at pH = 7 is

CCI(AB) = 11 - [pI(A)-7] [pI(B)-7] × 19/33.8

This formula gives an index between 1 and 20. The lowest index indicates the lowest possible attraction between amino acids (Asp-Asp) while the highest index indicates the highest possible attraction between amino acids (Arg-Asp). (In some cases it was convenient to move the range of CCI by -10.4 to give the neutral amino acid interaction a zero value (see 7).)

### Size compatibility matrix and index

There is a considerable variation in the sizes of amino acids (i.e. the length and bulkiness of the side chain residues, R). The molecular weight (MW) of an amino acid is roughly proportional to its size. Suppose that the residue size has some influence on the bending of a peptide chain and on the amino acid co-locations (convex fits to concave) or, to take an extreme situation, there is already size compatibility at a single residue level. Theoretically, there might be size complementarity between amino acids, similar to nucleic acid base pairs, where the sum of purine and pyrimidine bases is always the same. A size compatibility index and matrix (SCI) is constructed to test these hypotheses.

Amino acid MW varies between 57 (Gly) and 186 (Trp) or between 1 and 130 if only the weight of the residue is counted (-56 for the peptide backbone). This gives an average R weight ~61.5 or ~123 for average residue pairs. The deviation of a given amino acid pair from this average residue weight (RW) is calculated using the equation

SCI = 20-|[MW(A)+MW(B)-123] × 19/135|

This equation gives a maximal score (20) for amino acid pairs with a common RW = 123 and minimal score (1) for the Trp-Trp pair with maximal deviation from average (129 + 129 - 123 = 135). (In some cases it was convenient to move the SCI range by -16.2 to divide the co-locations into two equal groups (see 8).)

We have constructed many different variants of these indexes and matrices; one is called the SCH index and matrix, which means the sum of the SCI, CCI and HCI values.

A further useful index and matrix is the natural frequency index and matrix (NFM), which gives the calculated propensity of amino acid pairs if the co-locations occur randomly between two sequences each containing one amino acid per codon (i.e. 20 different residues, 63 altogether; this matrix is not shown).

### Tools

We have developed a JAVA program called SeqX to detect, visualize and analyze residue co-locations in and between protein structures [14]. Eighty different protein structures were taken from the protein structure database [15] and residue co-locations were collected and summarized. This collection of 20 × 20 amino acid pairs is referred to as "SeqX 80" data. Two residues were regarded as co-located if at least one atom belonging to a residue was within 6 Å radius from the C_1alpha _atom of the other residue. Residue neighbors (± 5) located on the same sequence were excluded.

There are about 40 high quality collections of amino acid collocation data. A classical collection is from Miyezawa and Jernigan [16,17]. The numbers of amino acid contacts, as well as Contact Energies, showed an excellent correlation with the "SeqX 80" data (p < 0.0001, n = 210, linear regression analysis). This supports the general validity of the results.

A modified version of a dot-plot program, called Dotlet [16,18], was used to reconstruct residue co-locations from the primary protein sequences and different compatibility matrices. This program routinely uses different standard matrices (such as PAM and Blosum) and the modification made it possible to add any additional large 27 × 27 numerical matrix.

Student's *t*-test and linear regression analyses were used for statistical evaluation of the results.

## Results

**Figure 1:**
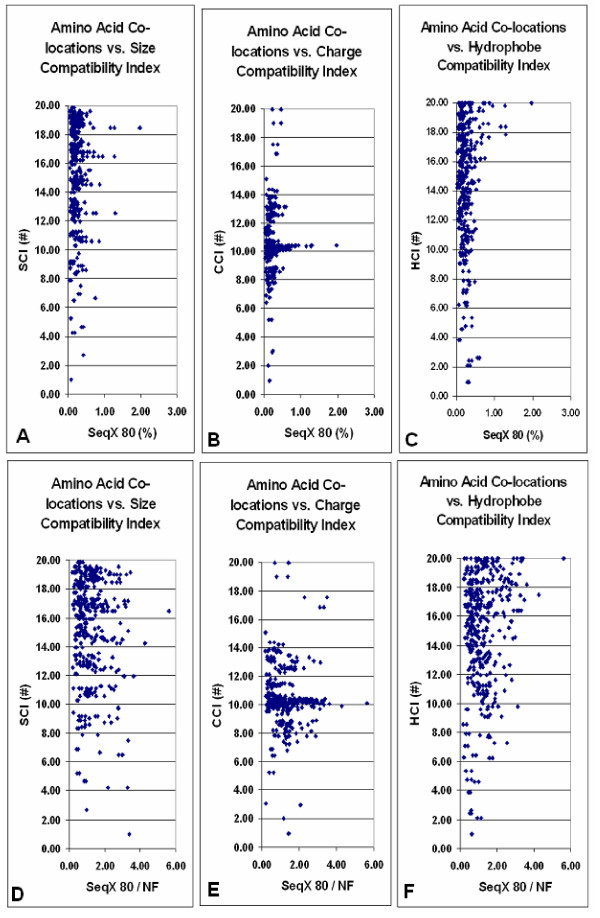
Amino acid co-locations vs. size, charge, and hydrophobe compatibility indexes. Average propensity of the 400 different amino acid co-locations in 80 different protein structures (SeqX 80) are plotted against size, charge and hydrophobe compatibility indexes (SCI, CCI, HCI). The original "row" values are indicated in (A-C). The SeqX 80 values were corrected by the co-location values, which are expected only by chance in proteins where the amino acid frequency follows the natural codon frequency (NF) (D-F).

Amino acid co-locations in the SeqX 80 collection showed a triangle-like distribution when plotted against SCI and HCI, and a more Gaussian distribution against CCI. This distribution pattern remained unchanged even when the SeqX 80 values were corrected for the natural frequency of amino acids and amino acid co-locations (NF), i.e. with the values expected to occur only by chance (Fig. 1).

**Figure 2:**
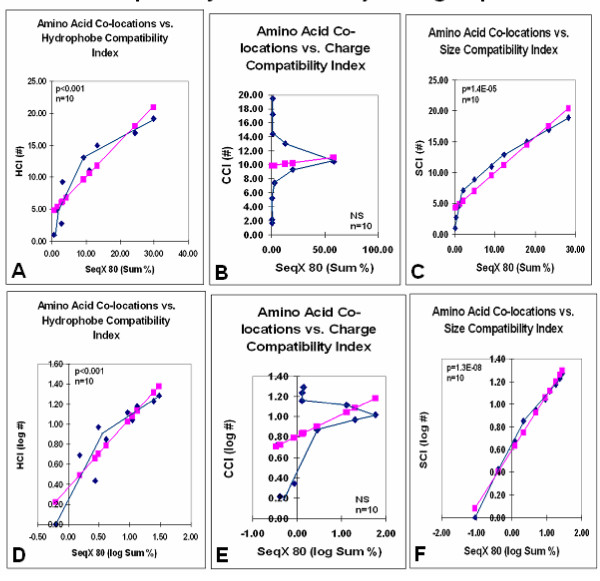
Amino acid co-locations vs. size, charge, and hydrophobe compatibility indexes in major subgroups. Data presented in Fig. 1 were divided into subgroups and summed (Sum). The group averages are connected by the blue lines while the pink symbols and lines indicate the calculated linear regression.

The detailed structure of these distributions suggested the presence of several subgroups within the size and charge compatibility distributions. The original data were therefore collected and summed into ten subgroups, each corresponding to two index units. Significant correlation was found for size and charge compatibility values, especially after logarithmic transformation; the charge compatibility distribution remained Gaussian and non-significant (Fig. 2).

**Figure 3:**
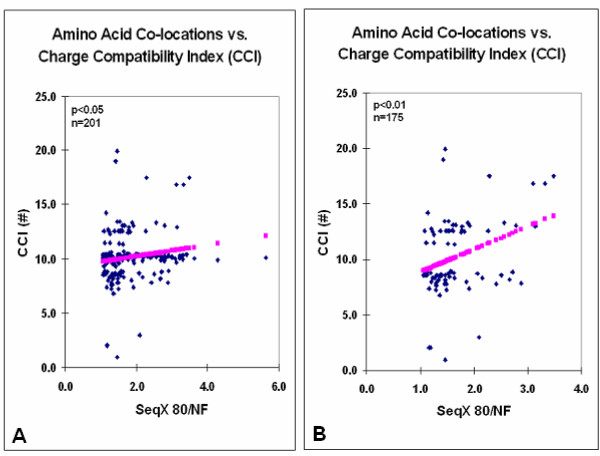
Amino acid co-locations vs. charge compatibility indexes after filtering for non-specific values. Data from Fig. 1E after removal of values <1 (SeqX 80/NF) (A) and belonging to co-locations between uncharged residue pairs (B).

The Gaussian distribution of the charge compatibility data in Figs. 1 and 2 seemed to be caused by a bulk of uncharged residue pairs, each having almost the same CCI values. The charge compatibility distribution became more similar to the size and hydrophobe compatibility distributions after the lowest scores (SeqQ 80/NF 0 to 1) were omitted, filtering the data for nonspecific values (Fig. 3).

**Figure 4:**
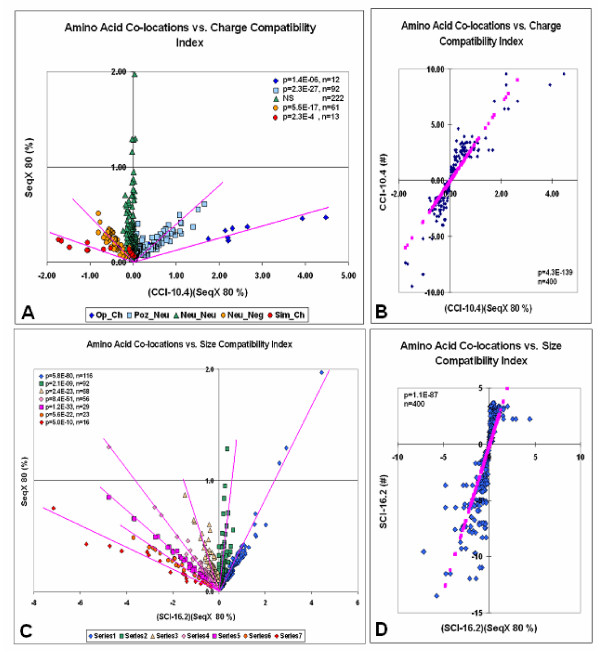
Amino acid co-locations vs. charge and size compatibility indexes. Weighted values. Index vs. SeqX 80 values are plotted against the weighted Index SeqX 80 values (i.e. index multiplied by the SeqX 80). This plotting method gave a clear separation of five different kinds of residue co-location (SeqX 80% values) regarding charge (Ch) compatibility (op, opposite; pos, positive; neg, negative; neu, neutral charges (A)) and seven different size compatibility (series 1–7 (C)). The linear regressions are indicated by pink lines. The correlation between the index values and the weighted Index_SeqX 80 values are indicated in (B) and (D). The pink symbols indicate the linear regression lines.

These results indicated that higher index values are often combined with higher co-location frequencies and the sum of higher scoring co-locations is more than the sum of the lower scoring co-locations. Therefore, multiplying the index with the co-location frequency is expected to multiply these differences. This method successfully separated five different subclasses of charge and seven different subclasses of size compatibility in residue co-locations (Fig. 4). The data for figure 4A and 4C were separated into different classes of interaction and were then fitted by regression. Finally, all data were reassembled as presented. The five subclasses of charge compatibility are in excellent agreement with the five possible types of interactions between charged residues: opposite, similar, positive-neutral, negative-neutral and neutral-neutral. We have not yet identified the differences among amino acids belonging to different size compatibility categories.

**Figure 5:**
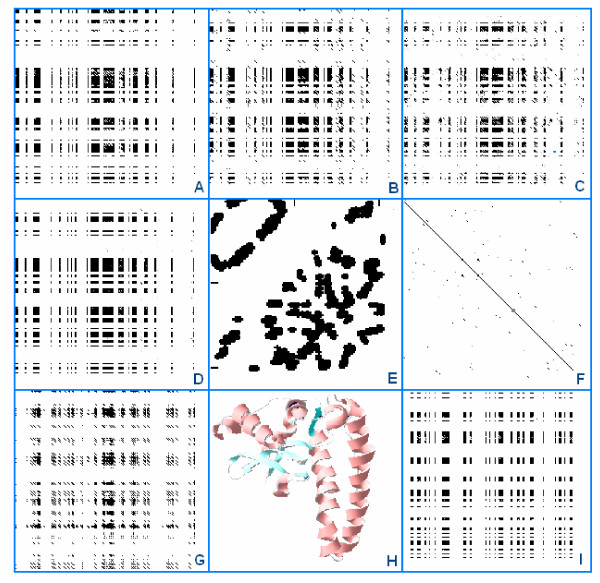
Matrix representation of residue co-locations in a protein structure (1AP6). A protein sequence (1AP6) was compared to itself with DOTLET using different matrices, SCM (A), CCM (B), HCM (C), the combined SCHM (D) and NFM (G) and Blosum62 (F). Comparison of randomized 1AP6 using SCHM is seen in (I). The 2D (SeqX Residue Contact Map) and 3D (DeepView/Swiss-PDB Viewer) of the structure are illustrated in (E) and (H). The black/gray parts of the dot-plot matrices indicate the respective compatible residues, except the Blosum62 comparison (F), where the diagonal line indicates the usual sequence similarity. The dot-plot parameters are otherwise the same for all matrices.

A modified version of the usual dot-plot method was suitable for locating compatible residues and subsequences. All three plus a combined matrix localized approximately the same residues, indicating that the three different kinds of compatibilities are represented by the same parts of the sequence. Randomizing the sequence or changing the matrix for a conventional Blosum matrix changed the pattern. The pattern produced by the NFM (the matrix consisting of the NF indexes and used as control) showed some distant similarity to the pattern obtained by SCHM. This might indicate that no one matrix is completely independent and distant from the natural frequency of amino acids in the proteins, which is of course determined by the number of synonymous codons per amino acid (Fig. 5).

**Table 1:**
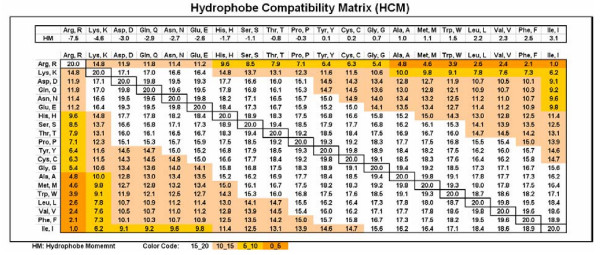
Table 1

**Table 2:**
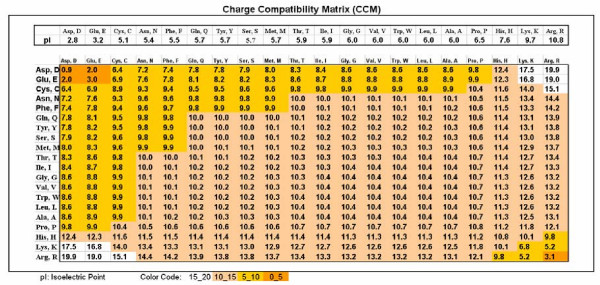
Table 2

**Table 3:**
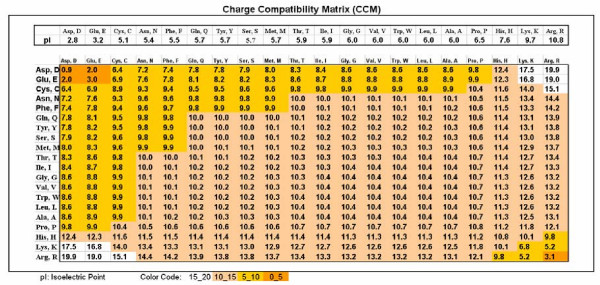
Table 3

I tried to reconstruct a simple protein structure from its sequence using the size, charge and hydrophobe matrices (Figure 5). It was not possible. It seems likely that the new matrices will play an important role in describing the correlation between physicochemical matrices and the 3D structure. An additional development is the prediction of different types of protein folds and the identification of patterns in the dot-plots that might act as signatures for structural folds at some SCOP level.

It was not possible to produce any dot-plot pattern resembling the original 2D or 3D view of the protein structure.

The overall patterns obtained by compatibility and similarity matrices seem to be fundamentally different. While similarity shows up in the dot-plot as a single diagonal line, the compatibility picture is more columnar with massive blocks and intersections. This seems to be consistent with the view that residue co-locations often occur in sequence-crossing sections rather than in linear alignments.

## Discussion

The first step of ab initio protein structure prediction (as well as protein design) is the prediction of the secondary structure (i.e. the location and length of alpha helices, beta strands and turns). This is a relatively easy task and several tools exist for the purpose. The next step is the further arrangement of the secondary structure elements into 3D, which usually involves sequence to sequence contacts between different parts of the peptide chain. Residue-residue contacts in and between peptide chains is not random; it is biased. Many indexes and matrices exist to describe it and much effort has been expended to connect these preferences to different physicochemical and biological circumstances, such as molecular configurations, intracellular locations, the structural or functional role of the protein, and even to different species, etc. [17-20].

Residue indexing is a relatively convenient method because it limits the number of possibilities to the number of the residue pairs.

It is believed that the main force that keeps a protein structure together is the hydrophobic interaction; many residues with the same hydropathy in one sequence interact with many residues with the same hydropathy in another sequence. The role of the powerful, but few, interactions between charges is probably much less. Size also plays a significant role in determining which parts of the structure fit to which other part; however, size and shape properties are often associated with larger protein domains formed by many residues (docking models). Size compatibility at residue level is well known from nucleic acid structures; there, the large purine bases always prefer interaction with the smaller pyrimidines. However, this type of size compatibility is not known to exist for individual amino acids.

The present observations confirm that hydropathy and charge properties do play an important role in determining residue co-locations in protein structures. Analysis of the results with the CCI indicated (not surprisingly) a higher prevalence of oppositely charged than similarly charged pairs. Less expected is the fact that positive-neutral pairs are significantly more prevalent (compatible) than negative-neutral pairs. This finding might further influence the role of charge in structure determination.

The SCI clearly indicates the presence of size compatibility in the amino acid bias: smaller residues preferentially co-locate with larger residues, while small-small and large-large co-locations are not preferred. This size preference at a single residue level is a novel observation.

Attempts were made to use the three different matrices in a dot-plot to predict the place and extent of the most likely residue co-locations. This semi-quantitative method indicated that the three very different matrices located very similar residues and subsequences as potential co-location places. No single diagonal line was seen in the dot-plot matrices, which is the expected signature of sequence similarity (or compatibility in our case). Instead, block-like areas indicated the place and extent of predicted sequence compatibilities. It was not possible to reconstruct a real map of any protein 2D structure.

This experience with the indexes provides arguments for as well as against Anfinsen's theorem. The clear-cut action of basic physicochemical laws at residue level is well in line with the lowest free energy requirement of the law of entropy. Furthermore, this obvious presence of physicochemical compatibility is easy to understand, even from an evolutionary perspective. In evolution, sequence changes more rapidly than structure; however, many sequence changes are compensatory and preserve local physicochemical characteristics. For example, if, in a given sequence, an amino acid side chain is particularly bulky with respect to the average at a given position, this might have been compensated in evolution by a particularly small side chain in a neighboring position, to preserve the general structural motif. Similar constraints might hold for other physicochemical quantities such as amino acid charge or hydrogen bonding capacity [21].

We were not able to reconstruct any structure using our indexes. However, there are massive arguments against Anfinsen's principle:

1. The connection between primary, secondary and tertiary structure is not strong, i.e., in evolution, sequence changes more rapidly than structure. Structure is often conserved in proteins with similar function even when sequence similarity is already lost (low structure specificity to define a sequence). Identical or similar sequences often result in different structures (low sequence specificity to define a structure).

2. An unfolded protein has a vast number of accessible conformations, particularly in its side chains of residues. Entropy is related to the number of accessible conformations. This problem is known as the Levinthal paradox [22].

3. The energy profile characteristics of native and designed proteins are different. Native proteins usually show a unique and less stable profile, while designed proteins show lower structural specificity (many different possible structures) but high stability [23].

4. The entropy minimum is a statistical minimum. The conformation entropy change of the whole molecule is the sum of local (residue level) conformation entropy changes and it permits the co-existence of many different local conformation variations. It is doubtful whether structural variability (heterogeneity, instability) is compatible with the function (homogeneity, stability) of a biologically active molecule.

The present experiments will not decide the "fate" of the Anfinsen's dogma; however, they show that the number of possible co-locating places is too large, and searching this space poses a daunting optimization problem. It is not realistic to expect the ab initio prediction of only one single structure from one primary protein sequence. The development of a prediction tool for protein structure (like an mfold for nucleic acids [4]), which provides only a few hundred most likely (thermodynamically most optimal) structure suggestions per protein sequence, seems to be closer. It is likely that SCM, CCI and HCM (or similar matrices) will be essential elements of these tools.

Additional folding information might be necessary (in addition to that carried in the protein primary sequence) to be able to create a unique protein structure. Such information is suspected to be present in the redundant genetic code [24-26] and chaperons [27-29].
